# Rapid early hematological response in a newly diagnosed patient with very severe aplastic anemia: a case report of high-dose romiplostim, hetrombopag and IST combination therapy

**DOI:** 10.3389/fmed.2026.1793166

**Published:** 2026-05-25

**Authors:** Liangkui Luo, Kaikai Huang, Yanbin Pang

**Affiliations:** Department of Hematology, Shenzhen Hospital of Southern Medical University, Shenzhen, China

**Keywords:** early response, hetrombopag, immunosuppressive therapy, romiplostim, very severe aplastic anemia

## Abstract

**Background:**

Very severe aplastic anemia (VSAA) is a life-threatening bone marrow failure syndrome. For patients ineligible for allogeneic hematopoietic stem cell transplantation (HSCT), effective treatments that induce rapid responses remain limited. This case report describes a young VSAA patient who achieved an exceptionally rapid trilineage response to first-line therapy with high-dose romiplostim (ROMI), hetrombopag, and standard immunosuppressive therapy (IST).

**Case presentation:**

A 16-years-old male with newly diagnosed VSAA (neutrophils 0.13 × 10^9^/L, platelets 11 × 10^9^/L) declined HSCT due to fertility concerns. He received ROMI (10 μg/kg weekly, titrated to 20 μg/kg), hetrombopag (7.5 mg daily), and IST (rabbit ATG 3 mg/kg/day for 5 days; cyclosporine 3 mg/kg/day). A partial trilineage response was achieved by week 9, with transfusion independence for red cells and platelets by week 12. By week 22, he achieved complete remission. After 7 months of follow-up, he remained in complete remission (neutrophils 2.2 × 10^9^/L, hemoglobin 127 g/L, platelets 160 × 10^9^/L). ROMI has been gradually tapered and is currently administered at 10 μg/kg per month. Throughout the treatment period, the patient experienced only grade 1–2 nausea and muscle spasms.

**Conclusion:**

The combination of high-dose ROMI, hetrombopag, and IST induced a rapid response in this young VSAA patient who refused HSCT. This dual TPO-RA regimen warrants further investigation as a potential first-line option for HSCT-ineligible VSAA patients.

## Introduction

Very severe aplastic anemia (VSAA) is a life-threatening bone marrow failure disorder. Allogeneic HSCT is curative but often unavailable (1). In real-world settings, the actual utilization rate of allo-HSCT is around 10% in regions with limited access to transplantation facilities and a lack of acceptable donors in registries (2). Immunosuppressive therapy (IST) remains the standard for most patients, but outcomes in VSAA are poor, with a 5-years overall survival of only 41.3%, which is significantly lower than that in SAA (66.6%) (3). Infection and hemorrhage are major causes of early death, highlighting the critical need for rapid treatment response.

Adding the thrombopoietin receptor agonist (TPO-RA) eltrombopag to IST has improved SAA outcomes, increasing response rates from 60%–65% to ∼80% and complete response (CR) rates from <10% to ∼40% (4, 5). However, in VSAA, eltrombopag plus IST has not shown significant early efficacy, with only 12% CR and 26% partial response within the first 3 months (4). A real-world study also indicates that eltrombopag plus IST does not improve early hematologic response rates in VSAA patients (6). Romiplostim (ROMI) binds the extracellular c-MPL domain, while hetrombopag targets the transmembrane domain, potentially offering synergistic effects on hematopoietic stem cell (HSC) proliferation (7, 8). We hypothesized that combining two TPO-RAs with different mechanisms might improve outcomes in VSAA.

Herein, we present a case of newly diagnosed VSAA in a young adult who received first-line therapy comprising IST, ROMI and hetrombopag, resulting in rapid hematologic recovery and early transfusion independence. These clinical findings suggest that early implementation of IST, ROMI and hetrombopag may offer significant therapeutic advantages, particularly in cases where HSCT is not feasible.

## Case presentation

A 16-years-old male presented with skin purpura and had no prior hematologic disorders or malignancy. Complete blood counts: neutrophils 0.13 × 10^9^/L, platelets 11 × 10^9^/L, reticulocytes 3.4 × 10^9^/L, hemoglobin 84 g/L. Bone marrow smear showed markedly reduced trilineage hematopoiesis with no megakaryocytes and no dysplasia. Cytogenetics, PNH clone testing, and FISH for MDS abnormalities were negative. The patient met VSAA diagnostic criteria. He was advised to undergo HSCT but declined due to concerns about long-term quality of life, particularly fertility and sexual function.

**FIGURE 1:**
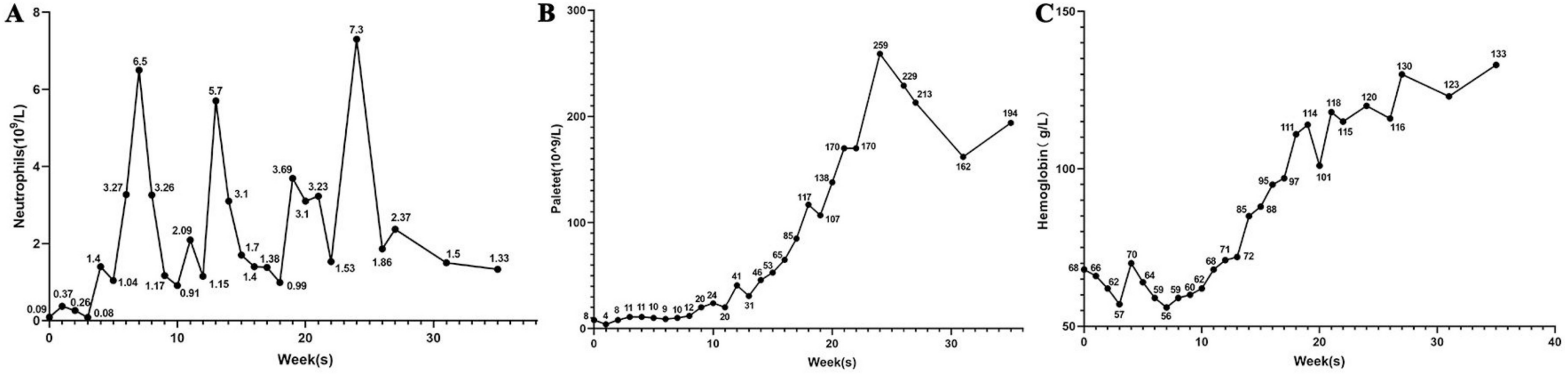
Longitudinal hematologic improvement in a patient following combined treatment with ROMI, Hetrombopag, and IST. **(A)** Neutrophils count; **(B)** platelets count; and **(C)** hemoglobin concentration.

**FIGURE 2:**
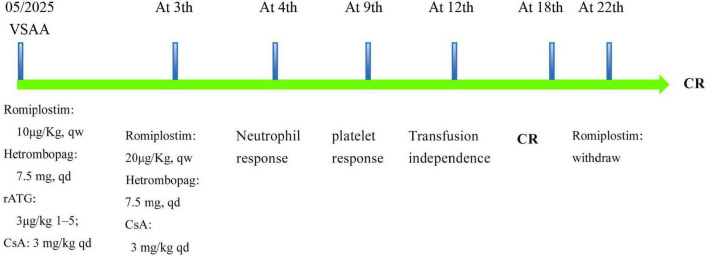
Timeline of patient’s diagnosis and treatment.

After discussing risks and benefits, treatment was initiated on May 13, 2025, consisting of: ROMI 10 μg/kg weekly (increased by 5 μg/kg weekly to a maximum of 20 μg/kg); hetrombopag 7.5 mg daily; rabbit ATG 3 mg/kg/day for 5 days; and cyclosporine A 3 mg/kg/day. At week 4, neutrophils were 1.40 × 10^9^/L (Figure 1A). At week 5, reticulocytes exceeded 20 × 10^9^/L. At week 9, platelets reached 20 × 10^9^/L (Figure 1B). Red blood cell transfusion independence was achieved at week 12 (hemoglobin 71 g/L) (Figure 1C). At week 18, the patient met CR criteria (neutrophils 1.00 × 10^9^/L, hemoglobin 111 g/L, platelets 117 × 10^9^/L). At week 22, after maintaining platelets > 150 × 10^9^/L for >4 weeks, ROMI was gradually tapered to 10 μg/kg per week, and hetrombopag was discontinued. He continued cyclosporine A. At 7-months follow-up (which was 6 weeks after ROMI discontinuation), CBC showed: neutrophils 2.2 × 10^9^/L, hemoglobin 127 g/L, platelets 160 × 10^9^/L, reticulocytes 61 × 10^9^/L. The patient continued to receive ROMI at a dose of 10 μg/kg monthly. During the treatment period, only grade 1–2 nausea and muscle spasms were observed; no grade 3 or higher adverse events, infections, or thromboembolism were observed. Figure 2 illustrates the treatment response over time. At the last follow-up, no adverse events (including but not limited to infection, thromboembolism or nephropathy) were observed. He is scheduled to return to school to continue his studies.

## Discussion

This case indicates that the first-line combination of high-dose ROMI (20 μg/kg), hetrombopag, and IST induced a rapid trilineage response in a young VSAA patient who declined HSCT. To our knowledge, this is the first report of such a dual TPO-RA regimen in pediatric VSAA, showing sustained remission even after early hetrombopag discontinuation.

For HSCT-ineligible SAA patients, IST has been the standard treatment regimen. Since 2014, TPO-RAs have shown significant efficacy. In a landmark trial, adding eltrombopag to IST improved 3-months CR from 10% to 22%. However, VSAA patients have poor early responses to eltrombopag-based regimens (12% CR at 3 months) (4). ROMI has shown promise in refractory AA. A dose-finding study reported that 10 μg/kg ROMI yielded a 70% platelet response rate at week 9 (9). Subsequent studies suggested that high-dose ROMI (10–20 μg/kg) combined with cyclosporine A achieved a 27-weeks response rate of 41.7% in treatment-naive patients (10). Importantly, in a cohort of 11 Eltrombopag-refractory AA patients, eight responded to high-dose ROMI, with 3 achieving trilineage responses (11).

Dual TPO-RA therapy may be more effective for the following reasons. ROMI acts on primitive HSCs via extracellular c-Mpl binding, while hetrombopag targets CD34+CD38+ committed progenitors through the transmembrane domain, providing potential synergy (12). Additionally, hetrombopag has iron-chelating properties that may enhance HSC sensitivity to ROMI (12, 13). The patient’s baseline reticulocytes (3.4 × 10^9^/L) and platelets (11 × 10^9^/L) were far below thresholds predictive of poor response (<37.77 × 10^9^/L and <10 × 10^9^/L, respectively), yet he achieved a rapid trilineage response, supporting a synergistic effect (14).

Limitations include the lack of specific Fanconi anemia testing (e.g., diepoxybutane or mitomycin C), though no suggestive physical findings or family history were noted. Also, rabbit ATG (used here) may be associated with slower early responses compared to horse ATG, making the observed rapid response even more notable.

## Conclusion

This case suggests that the front-line combination of high-dose ROMI, hetrombopag, and IST can induce rapid and sustained hematologic recovery in young VSAA patients ineligible for HSCT. The regimen was well tolerated. Further studies are warranted to validate this approach.

## Data Availability

The raw data supporting the conclusions of this article will be made available by the authors, without undue reservation.
